# Topological elastic liquid diode

**DOI:** 10.1126/sciadv.adt9526

**Published:** 2025-04-04

**Authors:** Yurong Zhang, Lijun Li, Gang Li, Zhen Lin, Ruteng Wang, Daobing Chen, Yifeng Lei, Di Tan, Zuankai Wang, Yan Zhao, Longjian Xue

**Affiliations:** ^1^ The Institute of Technological Sciences, School of Power and Mechanical Engineering, Wuhan University, South Donghu Road 8, Hubei, Wuhan 430072, China.; ^2^ Hubei Key Laboratory of Electronic Manufacturing and Packaging Integration, Wuhan University, Wuhan 430072, China.; ^3^ Department of Mechanical Engineering, Hong Kong Polytechnic University, Hong Kong 999077, China.; ^4^ School of Fashion and Textiles, Hong Kong Polytechnic University, Hong Kong 999077, China.

## Abstract

On-demand liquid transportation is fundamentally important and holds great potential in various fields, such as water collection and biological engineering. However, it remains highly challenging to in situ manipulate the direction of liquid flow on a lyophilic surface. Here, a topological elastic liquid diode (TELD) that could manipulate the flow direction is developed by combining the *Araucaria* leaf inspired ratchet array and the elasticity of silicon rubber. The flow pathway on the lyophilic TELD can be conveniently managed by regulating the competition forces along orthogonal directions at the liquid front, which is instantly realized by adjusting the mechanical strain in TELD (mode 1 regulation) or inserting extra forces at the liquid front (mode 2 regulation). Furthermore, TELD can serve as a logic gate, stress valve, microfluidic reactor, and fog collector. Thus, the work here establishes strategies for in situ and instant manipulation of liquid flow on a lyophilic surface.

## Teaser

A bioinspired topological elastic liquid diode is developed, being able to manipulate the direction of lyophilic flow on it.

## INTRODUCTION

On-demand transportation of liquid flow is highly desired in a wide range of technical systems such as microfluidics (*1*, *2*), inkjet printing (*3*, *4*), biomedical engineering (*5*, *6*), and so forth. Spontaneous and directional transportation of liquid is a key requirement for these applications, and the corresponding phenomenon has been observed in various natural living organisms (*7*–*13*), such as desert beetle *Stenocara*, cactus *Opuntia microdasys*, and *Crassula muscosa*. On these biological surfaces, liquid tends to flow toward regions with a smaller curvature, higher surface energy, or less pinning effect, driven by the difference in Laplace pressure, capillary force, or dynamics of liquid spreading. Mimicking these natural surfaces, synthetic surfaces with gradients of chemistry and/or topography have been constructed (*14*–*19*). For instance, by combining liquid-like coatings and asymmetric wedge-shaped channels, Soltani and Golovin (*18*) achieved long-range transportation of low surface tension liquids along the designed route. On the surface composed of an array of U-shaped islands spatially confined in periodical fences, we developed a liquid diode capable of rapid, unidirectional, and long-distance liquid transportation (*19*). These passive strategies, however, suffer from limitations of real-time control of the movement of liquid flows.

On the other hand, active methods that use external stimulus—such as heat, light, ultrasound, magnetism or electric fields—have been used to break the wetting symmetry of a water droplet, facilitating the moving of droplet in the desired directions (*20*–*28*). For example, by locally heating one side of the droplet (or the substrate beneath), the moving of droplet can be precisely guided remotely by light (*28*). While these methods gained precise manipulation of droplets, they are restricted by the requirements of additive in the droplet or the substrate, small volume of drop, accelerated liquid evaporation, and low transportation velocity. Moreover, these strategies only work on the surfaces that are lyophobic (or even superlyophobic) to the droplet. Therefore, the realization of in situ direction regulation of a liquid flow on a lyophilic surface remains highly challenging.

**Fig. 1.**
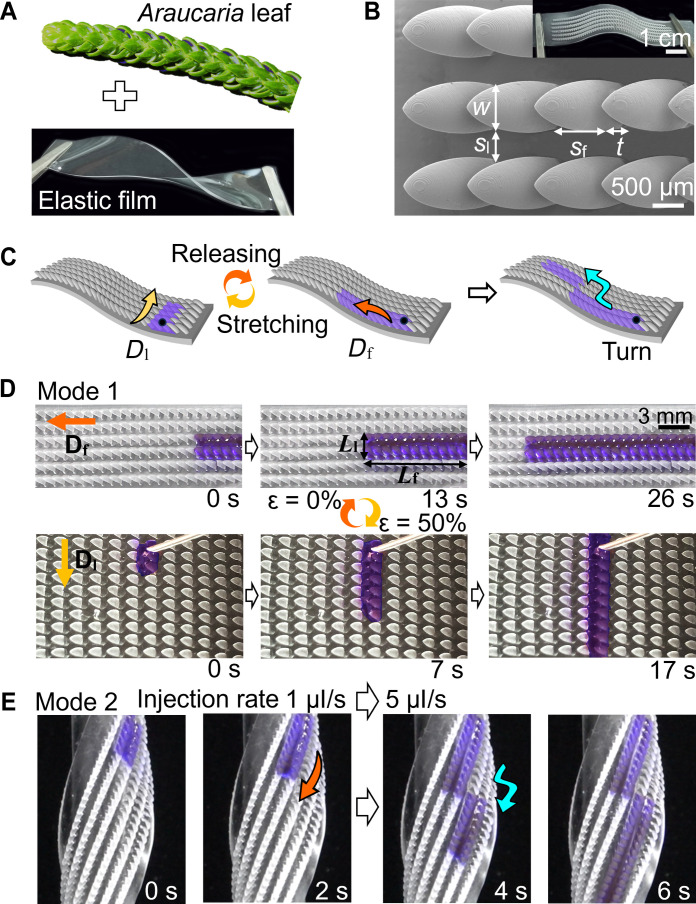
Topological elastic liquid diode. (**A**) Design and concept of TELD. (**B**) Scanning electron microscopy image of TELD with ratchet width (*w*) and space (*s*_f_ and *s*_l_) between ratchets indicated. The inset is an optical image of TELD. (**C**) Schematic of the capability of TELD to manipulate flow direction. (**D**) Optical snapshots showing the switching between forward direction (*D*_f_) (top) and lateral direction (*D*_l_) (bottom) of ethanol transportation at an injection rate of 1 μl/s on TELD with *k* of 1.6. The spreading length of ethanol along *D*_f_ (*L*_f_) and *D*_l_ (*L*_l_) are indicated. (**E**) On a helical TELD, the flow direction is manipulated by instantly changing the injection rate from 1 to 5 μl/s.

Here, we develop a topological elastic liquid diode (TELD) having the ability of in situ and real-time regulation of liquid flow pathway, along with the capability of unidirectional and long-distance liquid transportation on a lyophilic surface (Fig. 1A). The on-demand control of liquid flow on TELD is achieved by regulating the competition of forces along orthogonal directions via two modes, mechanical strain in the elastic supporting layer or the injection rate of flow. We envision that TELD holds the potential to revolutionize fluid control by offering enhanced versatility and adaptability in fluid transportation process and will offer a new perspective in scientific research, industrial technology, and biomedical application.

## RESULTS

### Design, fabrication, and function of TELD

Inspired by the ratchet array in *Araucaria* leaf (*10*), TELD was designed and constructed using polydimethylsiloxane (PDMS) (Fig. 1A). Replicated from the three-dimensional (3D) printed ratchet array, a soft negative mold composed of PDMS was constructed. Afterward, a PDMS film, uniaxially stretched to a predefined elongation, was covered onto the PDMS precursor-filled soft mold. After curing and releasing from the soft mold, TELD was obtained (Fig. 1B and fig. S1). The resulted PDMS has an elastic modulus of 1.18 ± 0.18 MPa and showed good elasticity (fig. S2). TELDs with various pre-elongations have identical ratchets with tilting angle α = 40°, length *l* = 1245 μm, thickness *t* = 300 μm, and width *w* = 650 μm (fig. S3). A structure coefficient, *k* = (*w* + *s*_l_)*/*(*t* + *s*_f_), where *s*_f_ and *s*_l_ are the space between ratchets along the ratchet-tilting direction (noted as the forward direction *D*_f_) and lateral direction (*D*_l_), respectively, that was used to define the TELD (Fig. 1B and fig. S4).

To demonstrate the capability of TELD in flow-direction manipulation (Fig. 1C), ethanol (surface tension γ = 22.3 mN/m) was used as the model liquid as it has a static contact angle (θ = 30.7° ± 1.2°) and advancing angle (θ_a_ = 35.5° ± 2.3°) on PDMS. While ethanol could slightly swell PDMS, the swelling has ignorable influence on its wettability on PDMS (fig. S5). Ethanol was continuously delivered onto the TELD by a pump at a certain injection rate. Unless indicated otherwise, the injection rate was 1 μl/s. On unstretched TELD with a *k* value of 1.6, ethanol dominantly propagated along *D*_f_, where the spreading length along the forward direction (*L*_f_) was much larger than that along the lateral direction (*L*_l_ along *D*_l_) (Fig. 1D and movie S1). When a tensile stress was applied to TELD, reaching an elongation (ε) of, such as 50%, the propagation of ethanol turned to *D*_l_ (Fig. 1D and movie S2). The releasing of stress would then reverse the flow to *D*_f_. The reversible regulation of flow direction was realized by the strain manipulation in the supporting layer of TELD, which is considered as the manipulation mode 1.

The manipulation of flow direction can also be achieved by instant changing of the flow rate. On a helical TELD, ethanol flowed along *D*_f_ within two columns at an injection rate of 1 μl/s. When the injection rate was suddenly increased, for example, to 5 μl/s, the flow track instantly shifted to the neighboring two columns and remained the flow direction along *D*_f_ (Fig. 1E and movie S3). In this case, the follow direction changed twice upon a single increasing of flow rate, which is considered as the manipulation mode 2.

### Mechanism and pathway manipulation of mode 1

**Fig. 2.**
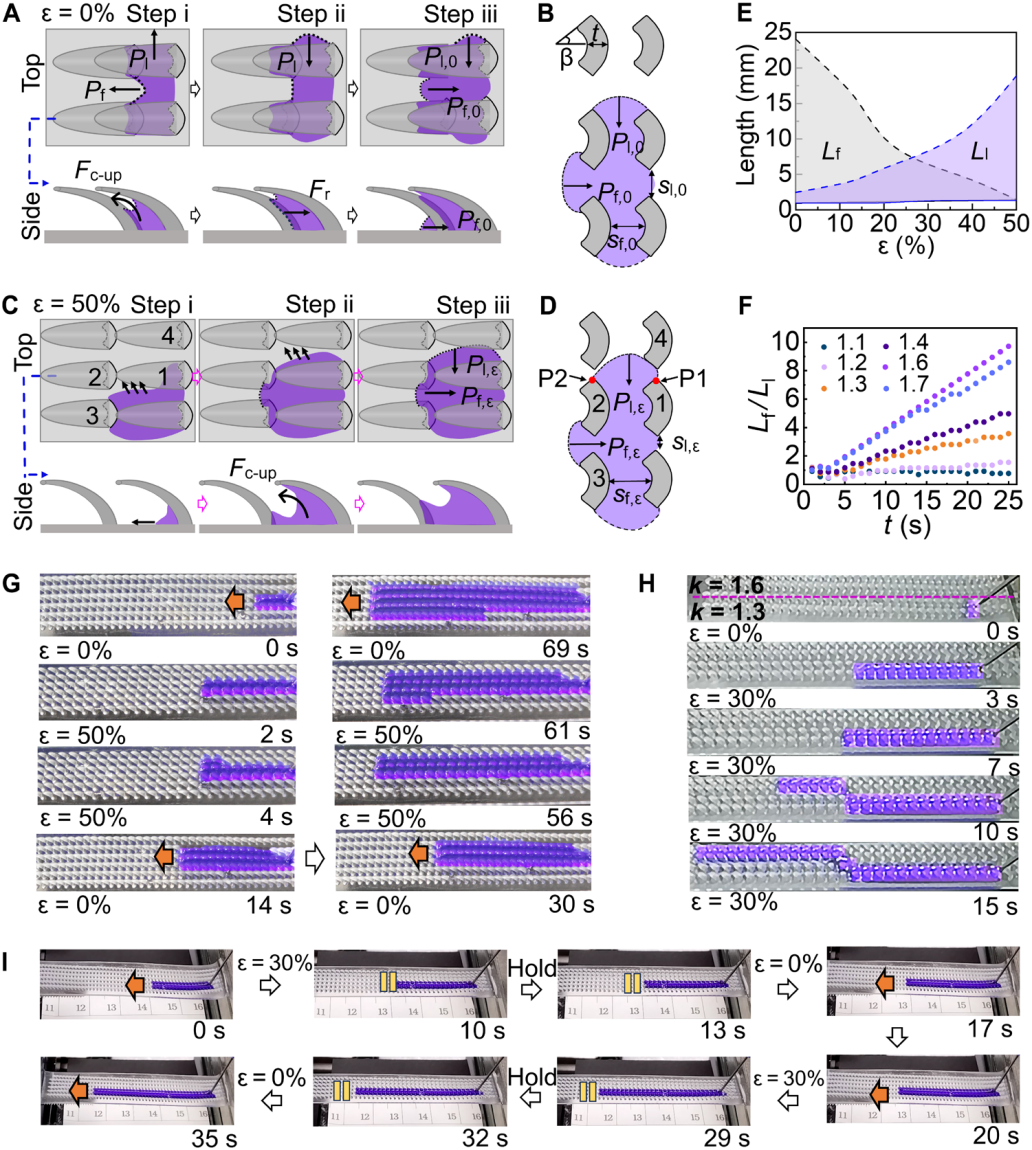
Mechanism and pathway manipulation of mode 1. (**A** and **C**) Schematic illustrations of top and side views of the three-step transportation of ethanol (dyed with purple) on (A) unstretched and (C) stretched (ε = 50%) TELD. (**B** and **D**) Schematic diagram of resistances at liquid front on substrate of TELD in step iii in (A) and (C), respectively. In (D), the numbers of ratchet are the same as that in (C). (**E**) Phase diagram showing the effect of elongation of TELD on *L*_f_ and *L*_l_. (**F**) Time sequences of the directional transportation capability (defined by *L*_f_/*L*_l_) of TELDs with different *k* values at an injection rate of 1 μl/s. (**G**) Sequential snapshots of ethanol flow (dyed with purple) on TELD at injection rate of 1 μl/s upon substrate stretching and releasing. (**H**) Snapshots of ethanol on composite TELD at an injection rate of 4 μl/s. (**I**) “Stress valve” effect of TELD (*k* = 1.6). Ethanol flow (dyed with purple) was delivered constantly with an injection rate of 1 μl/s. The orange arrow indicates the flow direction, and the yellow pause symbol represents the position holding the ethanol flow.

The directional transportation of liquid can be programmed by regulating the competitive forces along orthogonal directions at the spreading front. Taking unstretched TELD with a *k* value of 1.6 as the example, the transportation along *D*_f_ consists of three steps (Fig. 2A and movie S4). Initially, ethanol is filled into the cavity between ratchets upon injection and is pulled toward the ratchet tips by capillary force *F*_c-up_ ~ 2(*s*_f_ + *r*_1_)γcosθ_a_, where the transverse curvature radius *r*_1_ = 400 μm (step i). The deformation of the liquid-air interface caused by gravity can be neglected due to the relatively small dimension of characteristic length of the cavity (*~*961 μm) compared with the capillary length of ethanol [λ_cap_ = (γ*/*ρ*g*)^1/2^ = 1697 μm], where ρ = 0.789 g/cm^3^ and *g* are the density of ethanol and gravitational acceleration, respectively. Meanwhile, the liquid front (along the forward direction) has a saddle shape (fig. S6), introducing a Laplace pressure (*P*) pointing along the *D*_f_ (*P*_f_) (Fig 2A, step i). *P*_f,0_, which is *P*_f_ at ε = 0% and scales as 4γsinθ_a_*/*(2*t*sinβ + *s*_l,0_) with β being the half central angle, offers the liquid the tendency to flow forward, but could not drive the liquid to flow.

The further delivery of ethanol and the strong pinning at ratchet edges change the curvature of ethanol fronts. As (*t* + *s*_f,0_) is smaller than row-to-row period (2*t*sinβ + *s*_l,0_), the Laplace pressure along *D*_l_ (*P*_l,0_), which scales as ~4γsinθ_a_*/*(*t* + *s*_f,0_), is much larger than *P*_f,0_. Therefore, when the liquid front at *D*_l_ changes from a straight line to convex, the liquid front at *D*_f_ changes from concave to a straight line (Fig. 2A, step ii). It worth mentioning that the side surface of liquid remained concave, which is believed to be originated from the strong pinning force *F*_r_ ~ 2*l*γsinαsin(θ + β) of ratchet edges (*10*). The further delivery of liquid also converts the liquid front at *D*_f_ into convex shape (Fig. 2A, step iii). It means Laplace pressures at both liquid fronts pointing toward the liquid bulk, inhibiting the further spreading of liquid front on the substrate (Fig. 2B). As *P*_f,0_ is much smaller than *P*_l,0_, the continuous injection of ethanol increases the pressure inside ethanol over *P*_f,0_ that the depinning happens at the front ratchet edges (fig. S7), releasing the local pressure and allowing ethanol spread onto the bottom side of the front ratchets. The concave shape of the bottom side of ratchets even accelerates the spreading because the concave surface is in favor of reaching θ_a_ for the liquid front (Fig. 2B). Meanwhile, the pressure inside liquid cannot reach *P*_l,0_, that the depinning will not happen along *D*_l_. These two effects thus drive ethanol to flow along *D*_f_. Once the liquid front reaches the next-row ratchet, the liquid will be pulled up by capillary, starting the next round of steps i to iii.

The stretching of TELD regulates the spacing between ratchets (fig. S8) and therefore the flowing direction of ethanol. When TELD with *k* of 1.6 is stretched to ε = 50%, the overlapping between ratchets along *D*_f_ is eliminated. Ethanol climbs up on the bottom side of ratchet 1 and spreads along *D*_l_ quickly due to the double-concave shape [Fig. 2C (step i) and movie S5], while the liquid front on the substrate spreads along *D*_f_ and *D*_l_ at relatively smaller speeds. The fast spreading on the double-concave surface pulls the liquid front into contact with ratchet 2. Meanwhile, the liquid front is pinned at point 1 on ratchet 1 before it reaches the edge of ratchet 2 (point 2). Upon the further delivery of ethanol, the forward liquid front fills the gap between the ratchets 2 and 3 (step ii). Because of the small gap between these ratchets and the strong pinning effect of ratchet sides, the convex shape of the liquid front between the ratchets 2 and 3 inserts a strong *P*_f,ε_ against the further spreading of the liquid front along *D*_f_. While the liquid front gets into contact with the top surface of ratchet 2, ethanol would not climb up on ratchet 2 but moves further along *D*_l_ following that on the bottom side of ratchet 1. As *P*_l,ε_ is smaller due to the much larger (*t* + *s*_f,ε_) as compared to (2*t*sinβ + *s*_l,ε_), liquid favors lateral spreading (Fig. 2D). Because of the asymmetric geometry of the liquid front, the further delivery of ethanol drives the part of liquid front at ratchet 1 to get into contact with ratchet 4 (step iii), initiating the step i in the next round.

That is, the competition between *P*_f,ε_ and *P*_l,ε_ determines the direction of liquid flow, which can be easily achieved by manipulating the strain in TELD (fig. S8). The critical ε is found to be around 27%, above which ethanol tends to flow along *D*_l_ (Fig. 2E). The pre-elongation of supporting layer during the fabrication process offers a large possibility to finely regulate the structural parameters in TELD, without the need of a series of templates with different structural parameters (*29*). Meanwhile, the directionality of liquid flow on TELD can be quantified by the ratio of *L*_f_/*L*_l_ (Fig. 2F). A *L*_f_/*L*_l_ > 1 means a strong capability of forward transportation along *D*_f_, while a ratio smaller than 1 indicates a tendency toward lateral flowing. *L*_f_/*L*_l_ increased following *k*, reaching its maximum value of 9.7 at *k* = 1.6 within 25 s. Along with a better capability of directional liquid transportation, the transportation speed also increased from 0.26 ± 0.01 to 0.96 ± 0.05 mm/s when *k* was increased from 1.1 to 1.6 (fig. S9). The further increase in *k*, however, slightly decreased the directionality and speed of liquid transportation. While the elastic modulus of PDMS has ignorable influence on the transportation speed of ethanol (fig. S10), the combination of PDMSs with different elastic moduli in the substrate, which could form a gradient structure in the resulted TELD, can gain programmed transportations in a single TELD (fig. S11). The transportation speed can be further regulated by changing the viscosity (fig. S12), surface tension (fig. S13), and injection rate (fig. S14) of the liquid. Moreover, TELD can be operated within a wide temperature window (fig. S15).

Therefore, flow pathway on TELD can be instantly regulated via strain in substrate. For instance, the stretching of TELD with *k* of 1.6 to ε of 50% stopped the forward flowing within two columns of ratchets and initiated the lateral spreading (Fig. 2G and movie S6). The release of stress reinitiated the forward flowing with flow width expanded to three columns. Upon further applying and releasing tensile stress, lateral and forward flows can be reversibly and repeatedly switched. It is worth mentioning that the flow width increased after the applying of tensile stress.

The flow pathway can be shifted with the flow width remained the same. For demonstration, a composite TELD composed of two parts with *k* values of 1.3 and 1.6 was used (Fig. 2H). Ethanol was initially transported forward on the part with *k* = 1.3 at an injection rate of 4 μl/s. When the composite TELD was stretched to ε = 30%, the flow shifted the pathway to the part with *k* = 1.6 after a 90° turning, which was followed by the forward flowing with same flow width (movie S7). When the composite TELD was stretched to 30%, *s* in the part with *k* = 1.3 was sharply increased to 1429 μm, much larger than that in the part with *k* = 1.6 (1250 μm) (fig. S8). That is, with ε = 30%, the flowing pattern at the part with *k* = 1.3 is lateral flowing, while that with *k* = 1.6 was forward flowing. Therefore, the flow shifted its pathway and kept its forward direction.

Using the interval of pathway shifting, the liquid flow on TELD can be instantly and in situ switched on/off by regulating the strain in the substrate of TELD. The ethanol flow, which flowed within two columns of ratchets on a TELD with *k* = 1.6, was used as the example (Fig. 2I and movie S8). Rapid stretching of TELD to ε of 30% stopped the liquid flow immediately (yellow pause symbol). Holding ε at 30% for 3 s suspended the liquid flow for the same period. Upon the release of stress (at 13 s), the liquid flow immediately restarted. Subsequent stress applying (at 20 s) and release (at 32 s) again switched the liquid flow off and on, respectively. Moreover, the liquid flow could be held for over 2 min (from 25 to 166 s) on TELD stretched to ε = 30%, although the liquid was continuously injected at a speed of 1 μl/s (fig. S16). During this process, no physical valve was used, only stress was applied. The effect is then named after “stress valve.” The applying of stress in substrate turns on the stress valve, blocking the liquid flow, while the release of stress turns off the valve, allowing the liquid to flow forward.

### Mechanism and pathway manipulation of mode 2

**Fig. 3.**
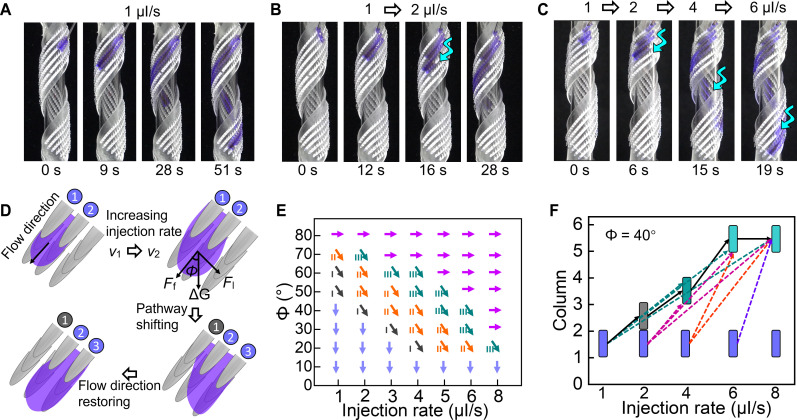
Mechanism and pathway manipulation of mode 2. (**A** to **C**) Pathway manipulation of ethanol flow on helical TELD by regulating the injection rate. Ethanol flow with injection rate (A) fixed at 1 μl/s, (B) changed from 1 to 2 μl/s at 12 s, and (C) changed from 1 to 2, 4, and 6 μl/s in sequence. The blue arrows in (B) and (C) indicate the location where the ethanol flow changed its pathway. (**D**) Schematic diagram showing the mechanism of pathway manipulation on helical TELD. (**E**) Influence of injection rate and tilting angle (Ф) on pathway shifting. The downward and rightward arrows indicate the forward and lateral flowing, respectively, while the diagonally downward arrow indicates the shifting of flow direction upon the increase of flow rate from 1 μl/s to the corresponding flow rate with the number showing the columns on TELD that ethanol flow shifted. (**F**) Pathway jumping of ethanol flow on helical TELD upon the changing of injection rate.

In mode 2, ethanol flowed along the initial pathway on a helical TELD, for instance, within two columns, when the injection speed (*v*) was constant (Fig. 3A). When the injection speed was suddenly increased, for instance, from original value of *v*_1_ = 1 to *v*_2_ = 2 μl/s, the track of liquid flow accordingly shifted to the neighboring two columns and continued the forward flow (Fig. 3B). The step increasing of injection speed from 1 to 2, 4, and 6 μl/s then laterally shifted the pathway thrice in sequence (Fig. 3C and movie S9).

The mode 2 manipulation on TELD was realized by inserting an extra force at the liquid front (Fig. 3D). When the injection speed is suddenly increased, ethanol cannot be transported to the in-front ratchets timely, resulting in increased ethanol accumulation at the flow front (fig. S17). The increased amount of liquid (∆*G*) can be estimated by ~*a*ρ(*v*_2_-*v*_1_)*pg*, where *p* is the period for speed changing and *a* is the prefactor proportional to *v*_1_. Depending on the tilting angle (Ф) of the TELD helix (Fig. 3E and figs. S18 and S19), the component of *∆G* along the lateral direction would add a driving force *F*_l_ = *∆G*sinФ ~ *a*ρ(*v*_2_-*v*_1_)*pg*sinФ to the lateral direction and a driving force *F*_f_ = *∆G*cosФ ~ *a*ρ(*v*_2_-*v*_1_)*pg*cosФ to the forward direction. The competitions between *F*_l_ and the resistance force *R*_p,l_ as well as between *F*_f_ and the resistance force *R*_p,f_ then determine the flow pattern upon the changing of injection speed (Fig. 3E and fig. S20). Once *F*_l_ could overcome the resistance force provided by *P*_l,ε_, the liquid front expands laterally and touches the ratchet in the neighboring column. The reaching of neighboring-column ratchet releases the accumulated liquid, which the ethanol would restore the flow direction, but under the new injection rate. Thus, the liquid flow keeps flowing in the forward direction along the new pathway. If the accumulated liquid cannot be compensated by reaching a new column, then two or more columns of ratchets may be involved in the lateral shifting of pathway (Fig. 3E). For instance, on TELD helix with Ф = 40°, when the injection speed was suddenly increased from 2 to 4 μl/s, the flow pathway was shifted from columns of one and two to that of three and four. Once the injection speed was sharply increased from 2 to 8 μl/s, the flow path jumped from columns of one and two to that of five and six (Fig. 3F). On the other hand, if *F*_l_ dominates, similar to that that at Ф = 80°, no matter how the injection speed is changed, ethanol remains flowing laterally (purple arrow in Fig. 3E).

### Application of TELD

**Fig. 4.**
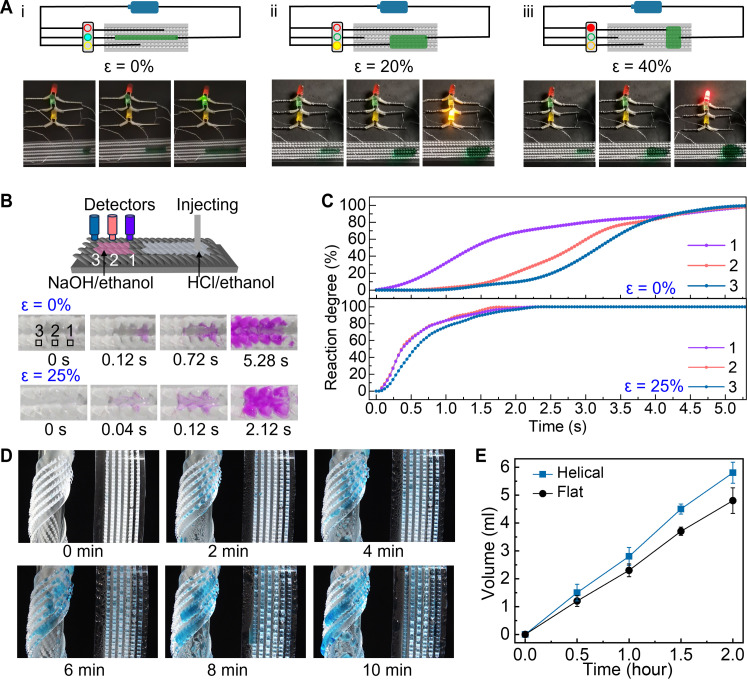
Application of TELD. (**A**) “Traffic light” regulation by stretching the TELD (*k* = 1.6) composed of three parts with (*w* + *s*_l_) values of ~1500, 1700, and 2100 μm to an elongation of (i) 0%, (ii) 20%, and (iii) 40%, respectively. The conductive solution is a mixture of ethanol, water, and NaCl with a mass fraction of 39:16:1. (**B**) Schematic showing the chemical reaction by delivery 1.5 M HCl/ethanol solution (stated with phenolphthalein) to NaOH/ethanol solution (6 μl, 5 mM) on TELD with positions 1, 2, and 3 on-line monitored by three individual detectors. (**C**) Variation of chemical reaction at positions 1, 2, and 3 on TELD with ε = 0% and ε = 25%. (**D**) Sequential snapshots of helical TELD and flat TELD collecting water in an environment with 85% relative humidity. (**E**) Capabilities of water collection of helical TELD and flat TELD. Data in (E) are the mean value of five measurements, and error bars are SD.

The capability to manipulate the flow pathway allows TELD to serve as a logic gate. For demonstration, a conductive solution was injected on the TELD, which was incorporated into an open-loop circuit. Under different stress, the conductive solution selectively connected to different wires, thereby turning on the corresponding light emitting diode (LED) (movie S10). Without applying stress to the TELD, the solution was transported straight forward along the preset two columns, turning on the green LED (Fig. 4A, i). An elongation of 20% of TELD expanded the liquid path to four columns, which turned on the yellow LED (Fig. 4A, ii). Further stretching to an elongation of 40% expanded the liquid flow to over six columns, switching on the red LED (Fig. 4A, iii). Therefore, the lighting of LED can be used to indicate the stress level in the TELD or in the object TELD attached to.

TELD can serve as a microfluidic reactor, combining the merits of traditional closed-channel microfluidics (*30*) and open droplet microfluidic system (*31*), having the advantages of small sample volume, rapid reaction, high flow rate, and the potential for integrating multiple on-site tests. Meanwhile, TELD offers extra functionalities as a mixer and controller. For demonstration, 1.5 mM HCl ethanol solution that contains phenolphthalein was transported toward the NaOH solution (6 μl, 5 mM) on TELD (Fig. 4B). The meeting of HCl solution with NaOH solution initiated the neutralization reaction and turned the base solution into red (movie S11). With three detectors, the reactions at positions 1, 2, and 3 were online monitored. While the reaction at position 3 started around 1.16 s later than that at position 1 due to the lateral displacement, the reaction processes finished almost at the same time (Fig. 4C, top row). When the TELD was stretched to ε = 25%, the distance between the three positions was enlarged. However, the reactions at these three positions almost started at the same time. Also, the reactions were also finished at the same time, within a much shorter period of around 1.92 s (Fig. 4C, bottom row). That is, TELD accelerated and homogenized the reaction. In addition, we believe that regulating the strain in TELD could further control the chemical reaction.

Making use of the force competition at the liquid front in mode 2, helical TELD showed a better capability of water fog collection than the flat TELD in an environment with 85% relative humidity. In front of humid flow, water droplets condensed on both TELD surfaces and merged together forming segmental liquid flows (Fig. 4D). The merging of neighboring segmental flows into liquid flows instantly added extra mass to the liquid front, similar to a sudden increase in injecting rate in mode 2, which shifts the flow pathway downward. Because of the limited width of TELD, the pathway shifting thus accumulated the liquid flows to the ratchet columns at the bottom, accelerating the flow velocity. In contrast, on the flat TELD, the liquid can flow forward only when the resistance force is overcome upon the accumulation of enough water at the liquid front. However, this accumulation effect does not present on the flat TELD as droplets are condensed randomly on the surface and that the accumulative effect does not exist. As a result, the efficiency of liquid collection was higher on the helical TELD than the flat TELD (Fig. 4E).

## DISCUSSION

In conclusion, inspired by the 3D ratchet in *Araucaria* leaf, TELD was constructed using a combination of 3D printing and soft lithography. TELD was demonstrated to have the capability of manipulating the pathway of directional ethanol flow in two modes. While ethanol flowed unidirectionally toward the tilting direction of ratchets, the flow direction can be turned 90° upon the mechanical stretching over a critical ε, such as 27% on TELD with *k* of 1.6. Stretching increases the resistance force along the forward direction and reduces that along the lateral direction, which in turn changes the flow direction (mode 1 regulation). The cycle of stretching and release thus reversibly altered the flow direction. Moreover, the competition of the resistance forces at orthogonal directions can also be regulated by inserting different forces in orthogonal directions at the liquid front, which was simply realized by changing the injection rate of ethanol on a helical TELD (mode 2 regulation). Depending on the increase in injection rate, the lateral shifting of pathway can be very well in situ manipulated. Furthermore, TELD was demonstrated to be able to serve as a logic gate, stress valve for liquid flow, microfluidic reactor, and fog collector. This work not only presents a strategy to control liquid flow on lyophilic surface but also proposes a flexible fluid transportation system with high potentials in the areas, such as soft electronics, laboratory on chip, and biological engineering.

## MATERIALS AND METHODS

### Materials

PDMS elastomer kit (Sylgard 184) and dimethyl silicone oils with different viscosities (0.65, 10, 100, and 200 cst) were purchased from Dow Corning. Ethanol, sodium hydroxide, hydrochloric acid, and sodium chloride were obtained from Sigma-Aldrich. Trichloro (1*H*, 1*H*, 2*H*, 2*H*-perfluorooctyl) silane and tetradecane were purchased from Shanghai Aladdin.

### Fabrication of 3D ratchets

Template of array of 3D ratchets (established with solidworks software) was transformed into white and black planar slices by using a slicing software Preform. The template was printed by curing the photopolymer resin (BMF, Micro Arch S230) layer-by-layer under ultraviolet light with a layer thickness of 5 μm.

### Fabrication of negative mold

The PDMS precursor was prepared by mixing the prepolymer and curing agent at a weight ratio of 10:1. After casting PDMS precursor onto the 3D ratchet template, air bubbles in PDMS precursor were removed in a vacuum desiccator for 30 min, which was followed by annealing at 90°C for 1 hour and demolding. After grafting trichloro (1*H*, 1*H*, 2*H*, 2*H*-perfluorooctyl) silane onto the surface, the soft negative mold was ready for use.

### Fabrication of TELD

The preparation of TELD can be divided into two steps. First, PDMS films were prepared by filling PDMS precursor (prepolymer and curing agent at a weight ratio of 10:1) into a glass template with a depth of 0.6 mm and cured at 90°C for 1 hour. The PDMS film was peeled off from the template and cut into 15 mm–by–50 mm rectangular strips. The PDMS strip was immobilized on a sit of clamps and uniaxially stretched to a predefined elongation. The PDMS precursor-filled negative mold was then brought into contact with the prestretched PDMS film and cured at 90°C for 1 hour. After demolding from the mold and releasing of the strain, TELD was ready.

Gradient PDMS film was achieved by filling PDMS precursor (prepolymer and curing agent at a weight ratio of 15:1) into a rectangular template with a width of 10 mm, a length of 10 cm, and a thickness of 0.6 mm. Then, PDMS precursors (10:1 and 20:1) were cast to the left and right side of the coated PDMS precursor (15:1). Redundant PDMS was removed using a glass rod. Upon curing at 90°C for 1 hour, gradient PDMS film was ready. Gradient TELD was then prepared following the procedure for normal TELD by using gradient PDMS film instead.

### Characterization

The morphology of the samples was characterized by field emission scanning electron microscopy (Sigma, ZEISS AG, Germany). Optical images of the samples and liquid transportation were captured by a digital camera (FDR-AX45, Sony, Japan). The mechanical properties of PDMS with various prepolymer–to–cross-linker ratios for TELDs were measured on a universal testing machine (Shenzhen Suns Technology, UTM2503, China) at a speed of 5 mm/min. The images of liquid transportation behavior on TELDs were acquired by an x-ray microscope in fast scan mode (XRM, Xradia 730 Versa, ZEISS, Germany). To study the influence of intrinsic wettability on liquid transportation, θ, θ_a_, and θ_r_ of ethanol mixed with water [the concentrations of ethanol from 0 to 100 weight % (wt %)] were detected by a contact angle goniometer (OCA25, Dataphysics, Germany) on a flat PDMS surface. The droplet volume for static contact angle measurement was 3 μl. θ_a_ and θ_r_ were evaluated by continuously injecting and retreating liquid with a volume up to 15 μl at a dosing rate of 1 μl/s. The average values of contact angles were acquired by measuring the same samples at least five different positions.

### Liquid transportation on TELD

The transportation behaviors of liquids with different ethanol concentrations on TELD were detected at room temperature. Ethanol mixed with water (0 to 100 wt %) was squeezed out of a stainless needle (with an inner diameter and outer diameter of 0.25 and 0.5 mm, respectively) at a rate of 1, 2, 4, 6, 8, and 10 μl/s using a liquid pump (LongerPump, LSP01-1A). To improve the visual clarity and the spatial resolution of ethanol spreading on samples, ethanol was dyed with oil red (5 mg/liter) or food dye (5 mg/liter) for pink or purple, respectively. The dynamic process of liquid transportation was documented by a digital camera from multiviews. In the experiment on the influence of temperature on the ethanol transportation behavior on TELD, temperature control (−50° to 60°C) was achieved by combining liquid nitrogen cooling stage and temperature controller.

### Pathway manipulation on TELD

To manipulate pathway on plane TELD, TELD was fixed on a displacement platform and stretched or released at a constant velocity of 4 mm/s. Continuous injection of dyed ethanol with a flow rate of 1 μl/s on the plane TELD surface was carried out during the whole test process. For the manipulation of ethanol pathway on helical TELD, TELD was fixed on a cylinder (with a diameter of 8 mm) at different tilting angles Φ, and the injection rate of ethanol was changed from 1 to 2 to 8 μl/s.

### TELD serves as a logic gate

An open-loop circuit, including TELD and three kinds of colored bulbs, was designed. The TELD (*k* = 1.6) composed of three parts with *w* values of ~1500, 1700, and 2100 μm. The conductive solution was injected on the TELD with a flow rate of 1 μl/s, which was a mixture of ethanol, water, and NaCl with a mass fraction of 39:16:1.

### TELD serves as a microfluidic reactor

A 1.5 mM HCl ethanol solution containing phenolphthalein was transported with a flow rate of 1 μl/s toward NaOH ethanol solution (6 μl, 5 mM) on TELD. The reactions at three different positions on TELD were detected simultaneously. The collected information was processed through an ImageJ software.

### Fog collection

Plane and helical TELDs, which have the same structural parameters and treated with oxygen plasma, were fixed vertically. Two glass graduated cylinders were placed beneath the TELDs. The water fog-laden air flow with a relative humidity of ~85%, which was generated by a humidifier, was guided to the TELD surfaces at the same time. The experiments were carried out at room temperature.
