# Supplementary material for: Topological elastic liquid diode
Source: Sci Adv. 2025 Apr 4;11(14):eadt9526. doi: 10.1126/sciadv.adt9526 (PMC11970454; doi:10.1126/sciadv.adt9526)
Supplement: Supplementary file 1 — Figs. S1 to S20 Legends for movies S1 to S11 [file sciadv.adt9526_sm.pdf]

Science Advances
AAAS

Supplementary Materials for

Topological elastic liquid diode

Yurong Zhang *et al.*

Corresponding author: Longjian Xue, xuelongjian@whu.edu.cn; Yan Zhao, yan2000@whu.edu.cn;
Zuankai Wang, zk.wang@polyu.edu.hk

Sci. Adv. **11**, eadt9526 (2025)
DOI: 10.1126/sciadv.adt9526

The PDF file includes:

Figs. S1 to S20
Legends for movies S1 to S11

Other Supplementary Material for this manuscript includes the following:

Movies S1 to S11

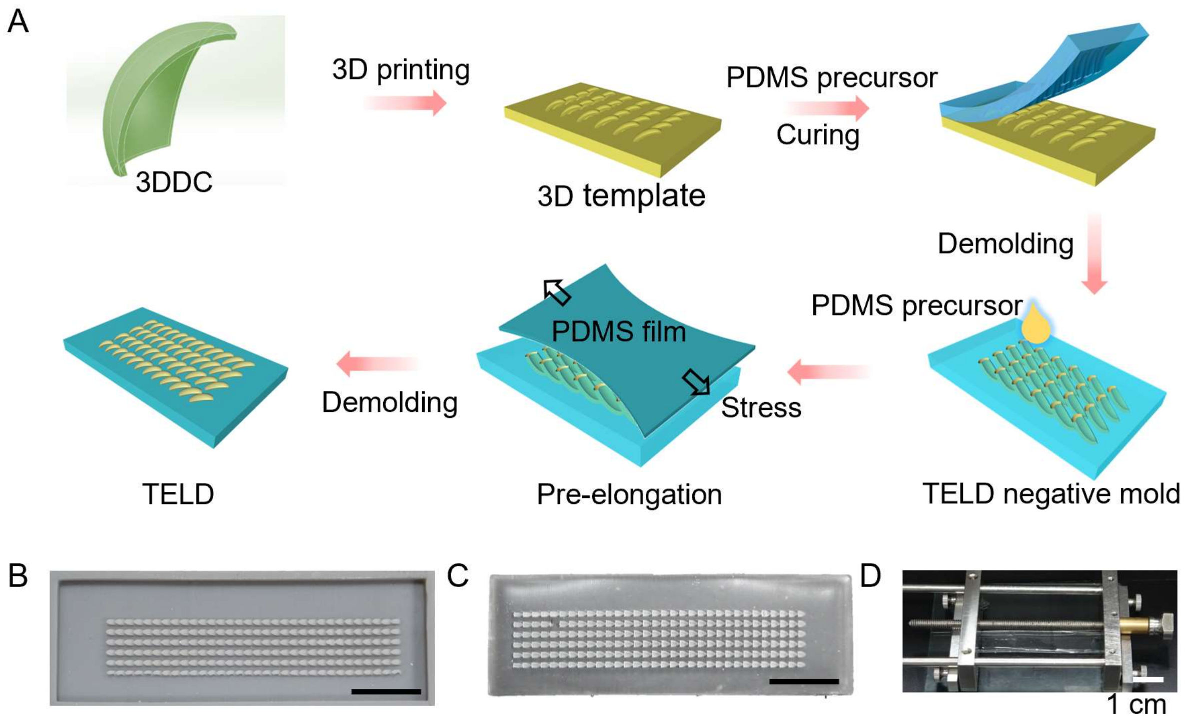

Fig. S1. Fabrication of topological elastic liquid diode (TELD). (**A**) Schematic illustration of the fabrication process of TELD. Optical image of (**B**) 3D-printed template and (**C**) PDMS negative mold. (**D**) The device and assembly of 40% pre-stretched PDMS film covered with a PDMS precursor-filled negative mold.

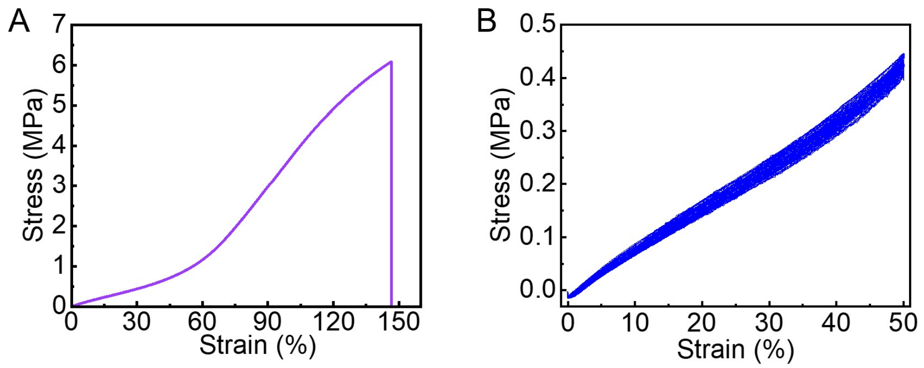

Fig. S2. Mechanical property of PDMS with mixing ratio of prepolymer to cross-linker being 10:1. (**A**) Typical stress-strain curve. (**B**) Cyclic stress-strain curve for 50 cycles.

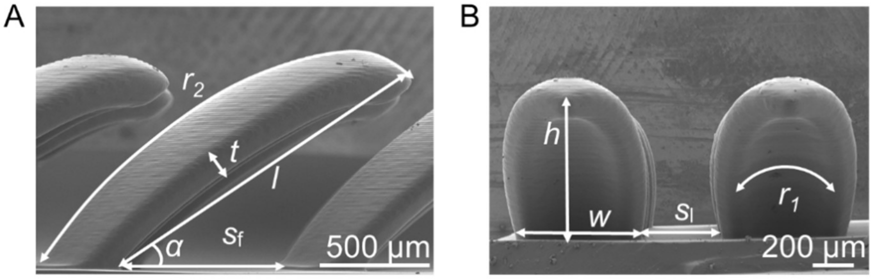

Fig. S3. Geometry of ratchet in TELD. Scanning electron microscopy (SEM) image of (**A**) side and (**B**) front view of ratchet with the radius of transverse curvature (r_1), radius of longitudinal curvature (r_2), tilting angle (α), thickness (t), height (h), width (w), space between adjacent rows (s_f) and adjacent columns (s_l) of ratchet are indicated.

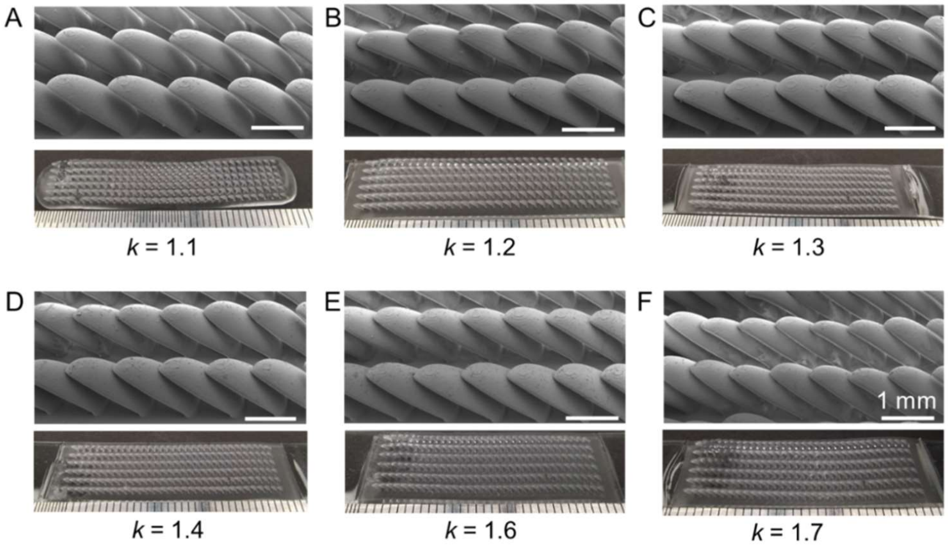

Fig. S4. Morphology of TELDs with different *k* values. SEM image (top row) and snapshot (bottom row) of TELDs with $k = (w + s_l)/(t + s_f)$ of (**A**) 1.1, (**B**) 1.2, (**C**) 1.3, (**D**) 1.4, (**E**) 1.6, and (**F**) 1.7, respectively.

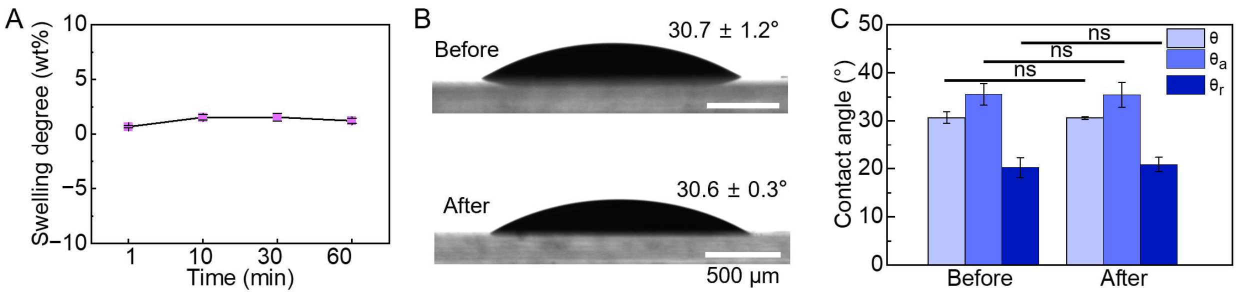

Fig. S5. Swelling of PDMS in ethanol and its impact on wettability. (**A**) Swelling of PDMS in ethanol. (**B**) Typical images and (**C**) static contact angle (θ) and dynamic contact angles (advancing angle θ_a, and receding angle θ_r) of ethanol on PDMS before and after swelling in ethanol for 20 h. One-way analysis of T-test, (ns: $P > 0.05$). Data in (A)-(C) are the mean value of 8 measurements and error bars are standard deviation.

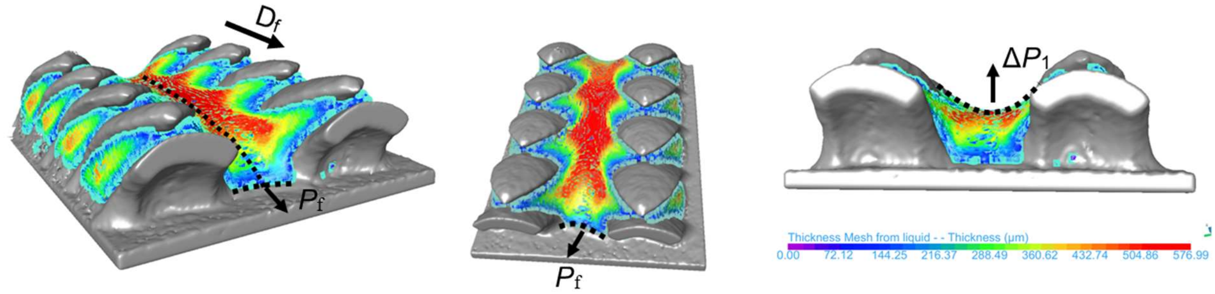

Fig. S6. Typical 3D images of GE solution (glycerol and ethanol with v:v = 1:5) on TELD, mimicking the state of ethanol on TELD (step i in Fig. 2A in the main text). Typical profiles are indicated by black dash lines.

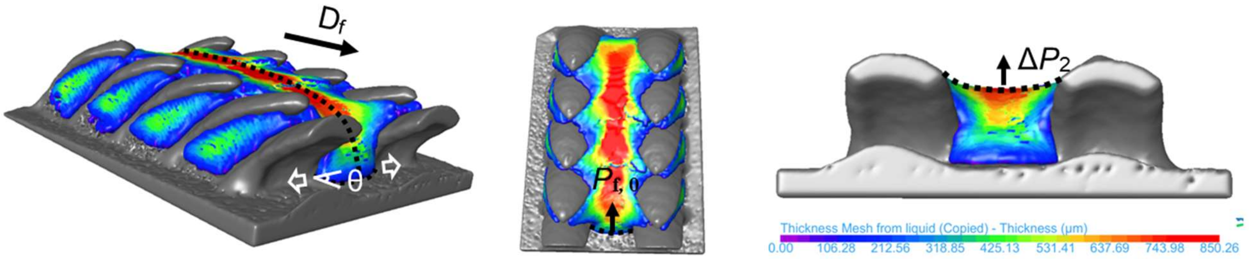

Fig. S7. Typical 3D images of GE solution on TELD, mimicking the state of ethanol on TELD (step iii in Fig. 2A in the main text). Typical profiles are indicated by black dash lines. The white hollow arrows indicate the tendency of liquid spreading.

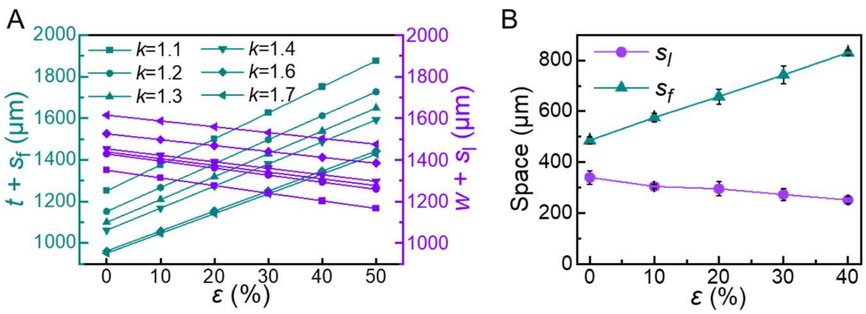

Fig. S8. Influence of elongation (ε) on structural parameters of TELD. The influence of ε on (**A**) ($t + s_f$) and ($w + s_l$) values of TELD with different k values and (**B**) ratchet pacing s_l and s_f of TELD with k =1.6, respectively. Data in (B) are the mean value of 5 measurements and error bars are standard deviation.

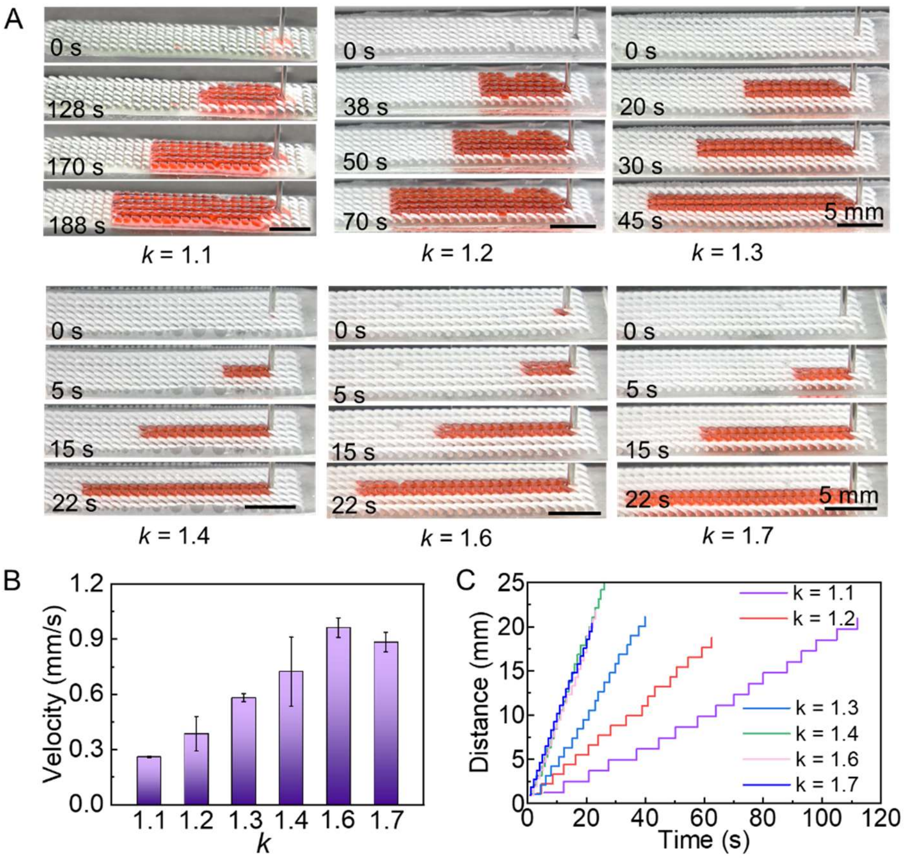

Fig. S9. Phenomenon of liquid transportation on TELD. (**A**) Snapshots of the ethanol transportation on TELD at an injection flow rate of 1 μL/s. Ethanol was dyed with oil red (5 mg/L). (**B**) Dependence of transportation velocity of ethanol on *k* of TELD. (**C**) Distances of unidirectional transportation of ethanol on TELDs at an injection rate of 1 μL/s. Data in (B) are the mean value of 6 measurements and error bars are standard deviation.

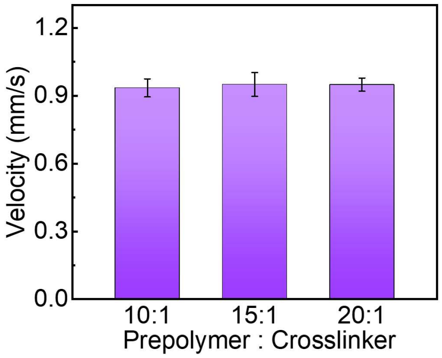

Fig. S10. Transportation speed of ethanol on TELDs with same structural parameters (k = 1.6) but various mixing ratios of prepolymer to crosslinker. Data is the mean value of 8 measurements and error bars are standard deviation.

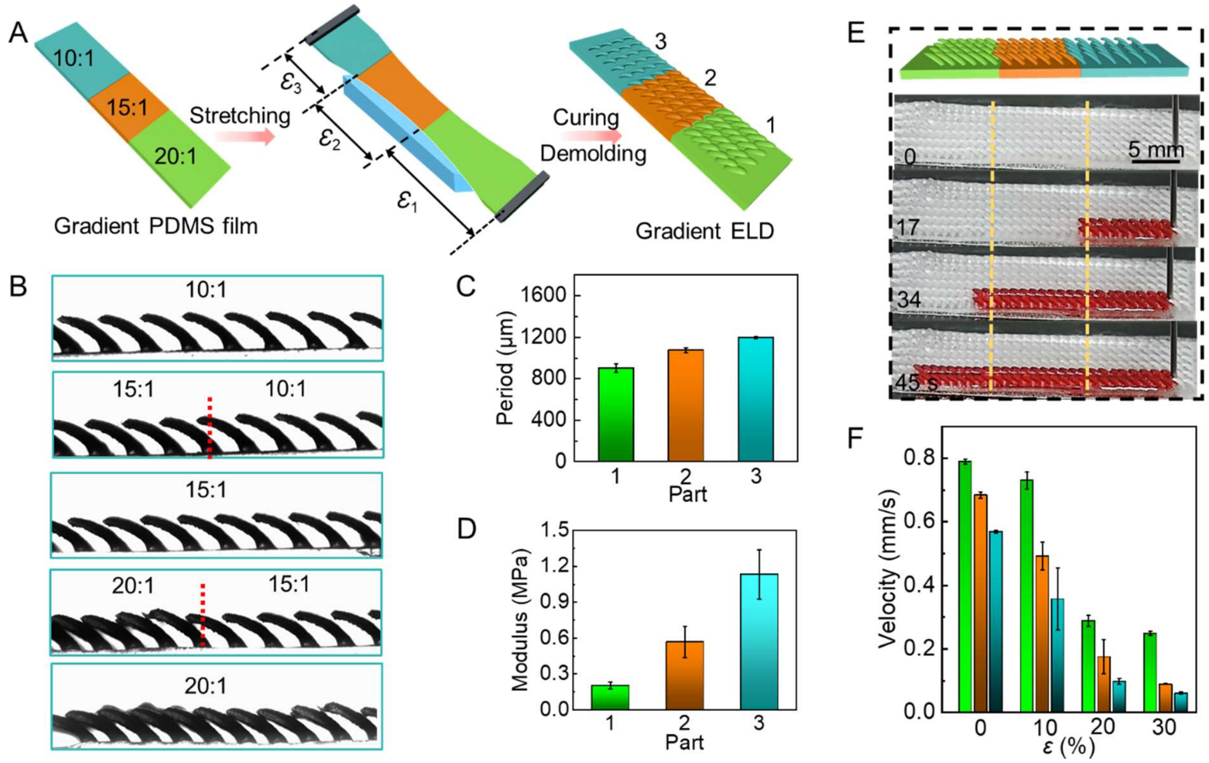

Fig. S11. Preparation and transportation capability of ethanol on gradient TELD. (**A**) Schematic illustration of the preparation of gradient TELD. The resulted parts of 1, 2 and 3 correspond to the parts with the mixing ratio of PDMS prepolymer to cross-linker being 10:1, 15:1 and 20:1, respectively. (**B**) Optical side views of gradient TELD at different positions with the border between different gradients marked with red line. (**C**) Period between ratchets on the parts of 1, 2 and 3 in gradient TELD. (**D**) Elastic moduli of different parts in the gradient TELD. (**E**) Cartoon and snapshots of ethanol transportation on gradient TELD. (**F**) Transportation velocity of ethanol flow on different parts of gradient TELD with various elongation under an injection rate of 1 μL/s. Data in (B, D, F) are the mean value of 5 measurements and error bars are standard deviation.

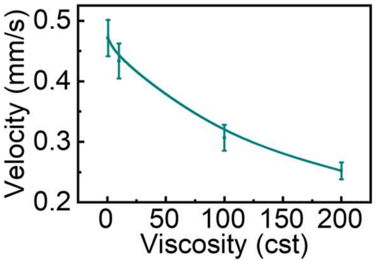

Fig. S12. Influence of liquid viscosity on the liquid transportation on TELD with *k* = 1.1. The liquids are dimethyl silicone oils with viscosities of 0.65, 10, 100, and 200 cst. Each data point is the mean value of 5 measurements and error bars are standard deviation.

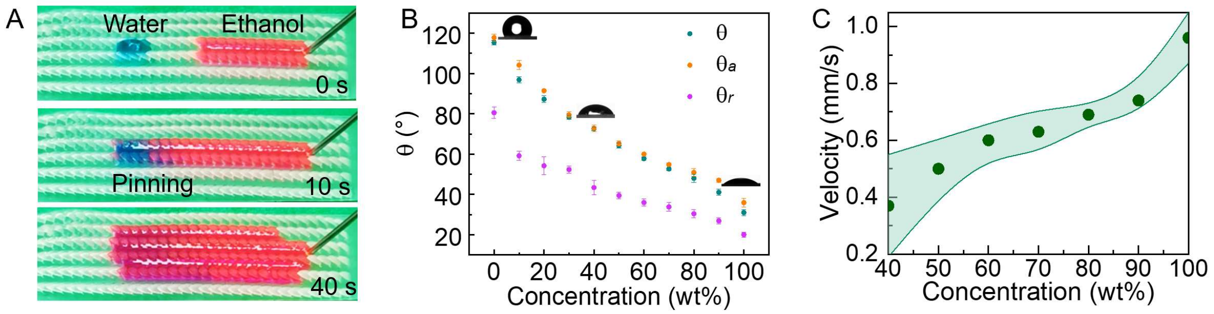

Fig. S13. Wettability of TELD. (**A**) A water droplet (6 μL, dyed blue) suspended the transportation of ethanol (dyed pink) on TELD. (**B**) Static contact angle θ, advancing angle θ_a and receding angle θ_r of ethanol-water mixture with concentrations of ethanol in water on a flat PDMS surface. (**C**) Transportation velocity of ethanol-water mixture with various concentrations of ethanol on TELD. Data in (B) are the mean value of 8 measurements and error bars are standard deviation.

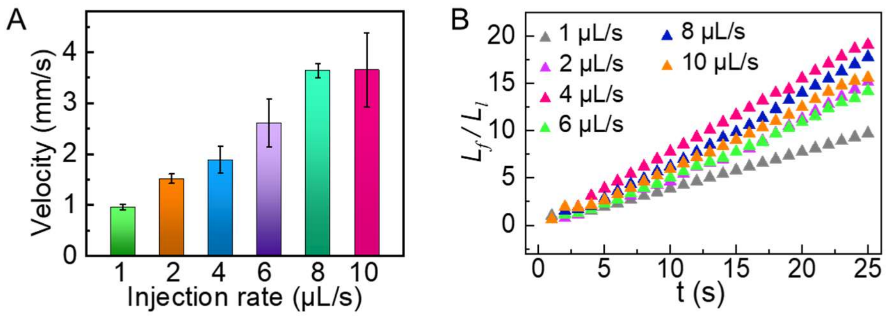

Fig. S14. Influence of injection rate on the liquid transportation on TELD with *k* = 1.6. (**A**) Dependence of transportation velocity of ethanol on the injection rate. (**B**) Time sequences of the directional transportation capability (L_f/L_l) of TELD with $k = 1.6$ at different injection rates. Data in (A) are the mean value of 6 measurements and error bars are standard deviation.

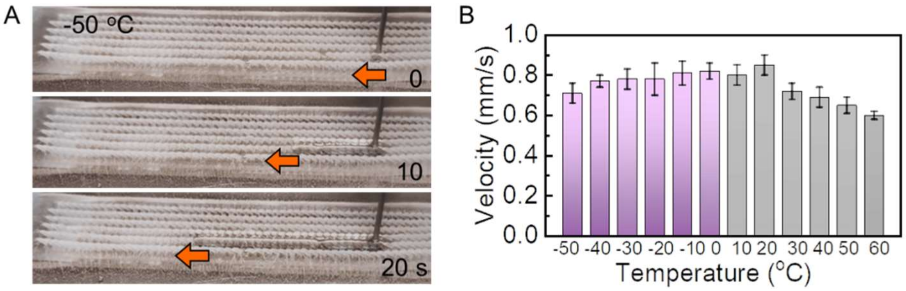

Fig. S15. The influence of temperature on the liquid transport on TELD. (**A**) Snapshots of the transportation of ethanol on TELD at -50 ºC. (**B**) Transportation velocity of ethanol on TELD with various temperatures. Data in (B) are the mean value of 6 measurements and error bars are standard deviation.

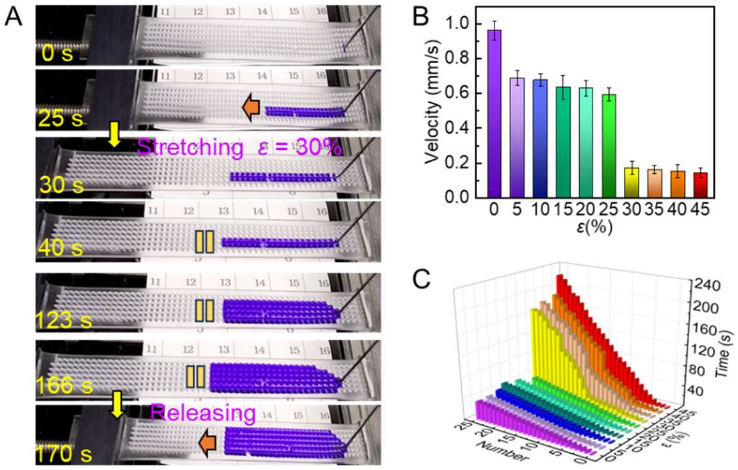

Fig. S16. Stress valve for TELD. TELD has a k of 1.6 and the injection flow rate was 1 μL/s. Ethanol was dyed purple. (**A**) Snapshots of ethanol transportation on TELD with stress applied to an $\varepsilon = 30\%$ applied at 25 s, followed by stress releasing at 166 s. (**B**) Dependence of transportation velocity of ethanol on ε in TELD. (**C**) Time required for ethanol flow to reach the sequential number of ratchets on TELD under elongations between 0 to 45%. Data in (B) are the mean value of 6 measurements and error bars are standard deviation.

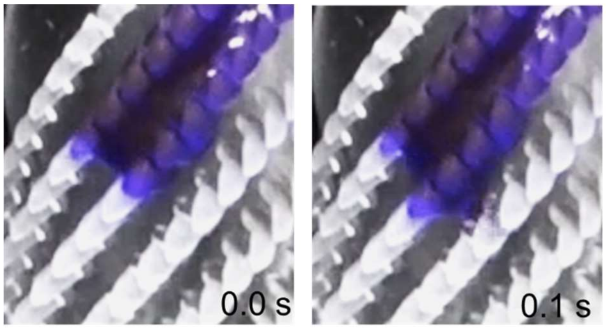

Fig. S17. Images of ethanol transportation on helical TELD with a tilting angle Φ of 40º. The injection rate changes from 1 to 2 μL/s.

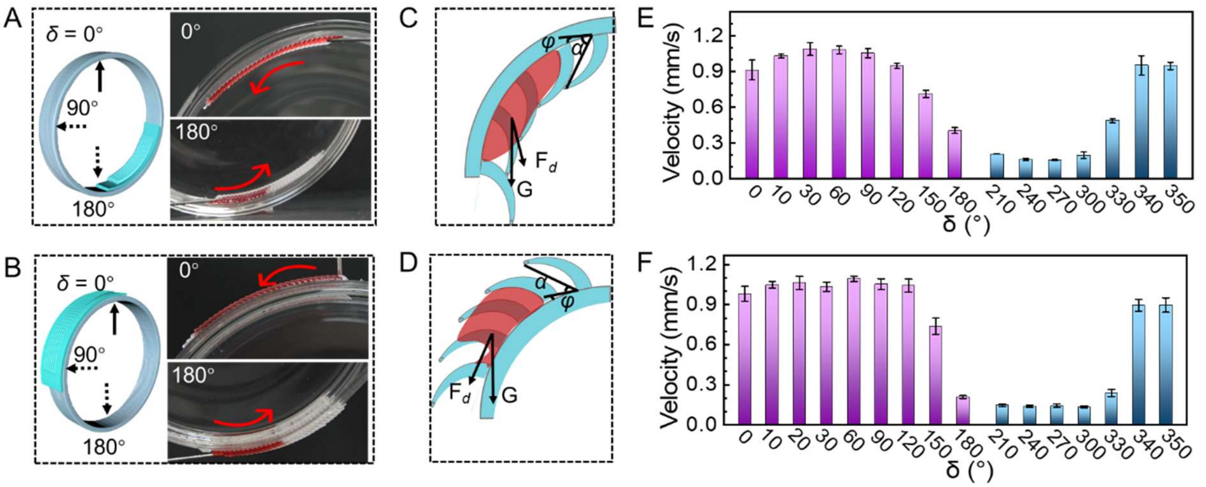

Fig. S18. Liquid transportation on curved TELDs. Schematic illustration of the position angle δ and the transportation process on the TELD with (**A**) positive curvature of 33.3 cm^{-1} and (**B**) negative curvature of -30.8 cm^{-1}, respectively. Red arrows indicate the flow direction on TELD with k = 1.6 at an injection rate of 1 μL/s. Ethanol was dyed with oil red. Schematic illustrations of forces on liquid on TELDs with (**C**) positive and (**D**) negative curvatures, respectively. Dependence of transportation velocity of ethanol on δ on TELDs with (**E**) positive and (**F**) negative curvature, respectively.

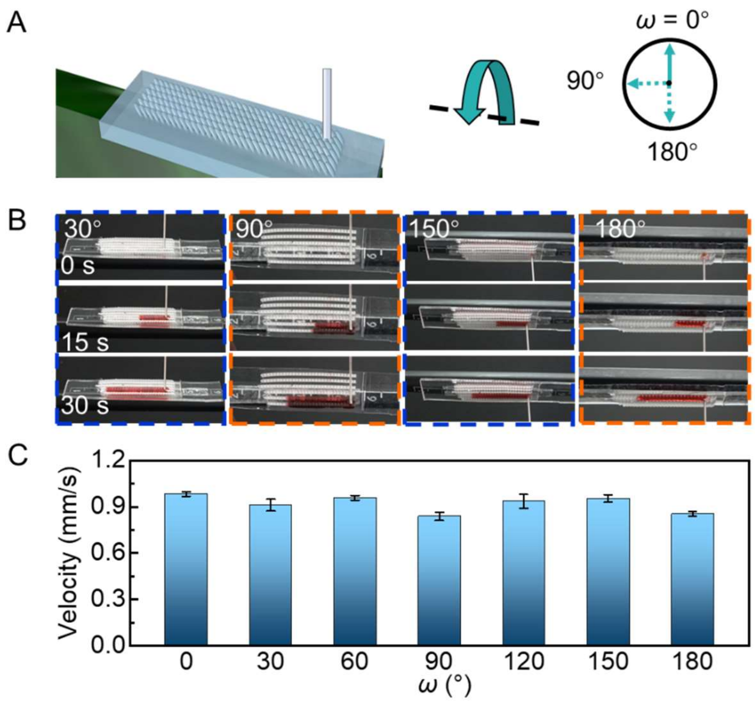

Fig. S19. Liquid transportation on inclined TELD. (**A**) Schematic illustration of the liquid transportation on TELD with various rotation angles, ω. (**B**) Snapshots of ethanol transportation on TELD inclined at ω of 30, 90, 150, and 180°. (**C**) Dependence of transportation velocity of ethanol on ω.

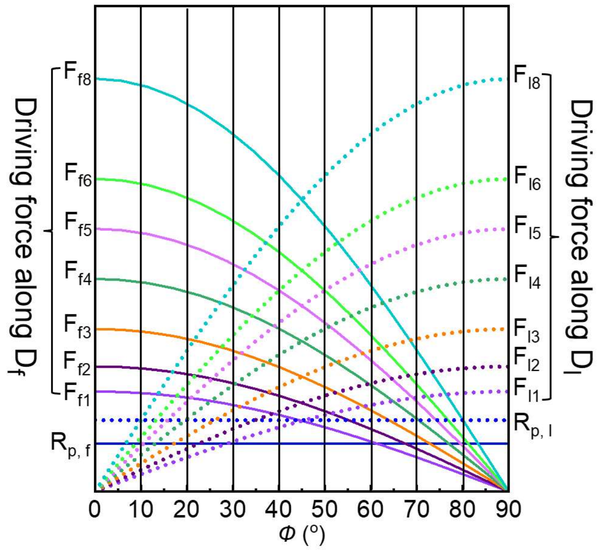

Fig. S20. Diagram showing the force competitions along D_f and D_l that determines the pathway shifting on helical TELD with various tilting angle $\boldsymbol{\Phi}$. F_{fn} (F_{ln}) are the driving force along D_f (D_l) at an injection rate of n μL/s. $R_{p,f}$ and $R_{p,l}$ are the resistance forces along D_f and D_l provided by the curved liquid front, respectively.

Supplementary Movies.

Movie S1

Ethanol transportation on TELD, corresponding to the data presented in Figure 1D. Ethanol was transported along the forward direction D_f on TELD with k = 1.6 at a constant injection rate of 1 μL/s.

Movie S2

Ethanol transportation on TELD, corresponding to the data presented in Figure 1D. Ethanol was transported along the lateral direction D_l on stretched TELD (ε = 50%) with k = 1.6 at a constant injection rate of 1 μL/s.

Movie S3

Liquid transportation on helical TELD with injection flow rate changed from 1 to 5 μL/s, corresponding to the data presented in Figure 1E.

Movie S4

Bottom view of transportation of ethanol on unstretched TELD (k = 1.6) with an injection rate of 1 μL/s, corresponding to the data presented in Figure 2A.

Movie S5

Bottom view of transportation of ethanol on stretched TELD (k = 1.6, ε = 50%) at an injection rate of 1 μL/s, corresponding to the data presented in Figure 2C.

Movie S6

Pathway manipulation of ethanol flow on TELD (k = 1.6) by mechanical stress in substrate, corresponding to the data presented in Figure 2G.

Movie S7

Pathway manipulation on composite TELD with k values of 1.3 and 1.6, corresponding to the data presented in Figure 2H.

Movie S8

Demonstration of "Stress valve" (the instant and in-situ on/off switching of liquid flow) effect of TELD, while ethanol (dyed purple) was continually injected at a speed of 1 μL/s. Movie corresponds to the data presented in Figure 2I.

Movie S9

Liquid transportation on helical TELD with injection flow rate step-by-step changed from 1 to 2, 4, 6 μL/s, corresponding to the data presented in Figure 3C.

Movie S10

Demonstration of "Traffic light" regulation on TELD. Conductive solution was injected with an injection rate of 1 μL/s, which was incorporated in an open loop circuit. The conductive solution is a mixture of ethanol, water and NaCl with a mass fraction of 39:16:1. Movie corresponds to the data presented in Figure 4A.

Movie S11

TELD is served as a microfluidic reactor, sequential reaction of 1.5 M HCl ethanol solution (added phenolphthalein) with NaOH ethanol solution (6 μL, 5 mM) on TELD with ε of 0% and 25%, respectively. The movie is corresponding to the data presented in Figure 4B.
